# Prediction of the Individual Aortic Stenosis Progression Rate and its Association With Clinical Outcomes

**DOI:** 10.1016/j.jacadv.2024.100879

**Published:** 2024-03-06

**Authors:** Constantijn S. Venema, Kees. H. van Bergeijk, Demetra Hadjicharalambous, Theodora Andreou, Jasper Tromp, Laura Staal, Jan A. Krikken, Hindrik W. van der Werf, Ad F.M. van den Heuvel, Yvonne L. Douglas, Erik Lipsic, Adriaan A. Voors, Joanna J. Wykrzykowska

**Affiliations:** a Department of Cardiology and Cardiothoracic Surgery, Heart Centre, University Medical Centre Groningen, University of Groningen, Groningen, the Netherlands; b Saw Swee Hock School of Public Health, National University of Singapore, and the National University Health System, Singapore, Singapore

**Keywords:** aortic stenosis, risk prediction, structural heart disease, transcatheter aortic valve replacement, valvular heart disease

## Abstract

**Background:**

The progression rate of aortic stenosis differs between patients, complicating clinical follow-up and management.

**Objectives:**

This study aimed to identify predictors associated with the progression rate of aortic stenosis.

**Methods:**

In this retrospective longitudinal single-center cohort study, all patients with moderate aortic stenosis who presented between December 2011 and December 2022 and had echocardiograms available were included. The individual aortic stenosis progression rate was calculated based on aortic valve area (AVA) from at least 2 echocardiograms performed at least 6 months apart. Baseline factors associated with the progression rate of AVA were determined using linear mixed-effects models, and the association of progression rate with clinical outcomes was evaluated using Cox regression.

**Results:**

The study included 540 patients (median age 69 years and 38% female) with 2,937 echocardiograms (median 5 per patient). Patients had a linear progression with a median AVA decrease of 0.09 cm^2^/y and a median peak jet velocity increase of 0.17 m/s/y. Rapid progression was independently associated with all-cause mortality (HR: 1.77, 95% CI: 1.26-2.48) and aortic valve replacement (HR: 3.44, 95% CI: 2.55-4.64). Older age, greater left ventricular mass index, atrial fibrillation, and chronic kidney disease were associated with a faster decline of AVA.

**Conclusions:**

AVA decreases linearly in individual patients, and faster progression is independently associated with higher mortality. Routine clinical and echocardiographic variables accurately predict the individual progression rate and may aid clinicians in determining the optimal follow-up interval for patients with aortic stenosis.

Aortic stenosis is one of the most common forms of valvular heart disease worldwide, and its prevalence is rapidly increasing due to longer life expectancy.^1^^,^^2^ Currently, the only available treatment for aortic stenosis is surgical or transcatheter aortic valve replacement (AVR), indicated when aortic stenosis has become severe and leads to symptoms or reduced left ventricular function.^3^ The latest European guidelines recommend follow-up every 2 to 3 years in younger patients with mild aortic stenosis and low aortic valve calcification, yearly in moderate aortic stenosis, and every 6 months in patients with severe aortic stenosis to timely identify patients with an indication for valve replacement.^3^ American guidelines are slightly less stringent and recommend follow-up every 3 to 5 years in mild aortic stenosis, every 1 to 2 years in moderate aortic stenosis, and every 6 to 12 months in severe aortic stenosis.^4^

However, the rate of progression may vary considerably between individual patients, and a fixed follow-up interval may be too long for one patient and too short for another. Yet, remarkably little is known about the individual progression of aortic stenosis over time.^5^^,^^6^

Therefore, we aimed to: 1) determine the average and individual rate of progression of aortic stenosis; 2) identify factors associated with faster progression; 3) determine whether faster progression is associated with worse clinical outcomes; and 4) develop a prognostic model to determine the individual rate of progression at initial echocardiographic diagnosis of aortic stenosis.

## Methods

### Study design and patient sample

This retrospective longitudinal cohort study was conducted at the University Medical Center Groningen, a tertiary referral hospital in the Netherlands. We performed a search query in the hospital’s electronic patient records to identify patients with moderate aortic stenosis between December 2011 and December 2022.^3^ Moderate aortic stenosis was defined as: aortic valve area (AVA) measured by continuity equation >1.0 and ≤1.5 cm^2^, peak jet velocity ≥3.0 and <4.0 m/s, and mean transvalvular gradient ≥20 and <40 mmHg. Patients identified by the search query who had 2 or more transthoracic echocardiograms available, at least 6 months apart, and conducted before AVR (if applicable), were included in this study. Patients with subvalvular aortic stenosis and congenital heart disease apart from bicuspid valve were excluded. The local research ethics committee approved this study, and the requirement for informed consent was waived (METc 2022/545). This study followed the Transparent Reporting of a Multivariable Prediction Model for Individual Prognosis or Diagnosis reporting guidelines for the development of prediction models.^7^

### Data collection

From each patient, we collected all available echocardiograms ranging from no aortic stenosis to severe aortic stenosis between December 2011 and December 2022 from the electronic hospital records. Older echocardiograms were excluded to reduce variability in imaging and reporting quality. Baseline (T = 0) was defined as the initial echocardiogram that showed at least mild aortic stenosis (peak jet velocity ≥2.6 m/s), as this would be the most crucial clinical indication to start monitoring progression. Thus, echocardiograms without aortic stenosis were coded as having a negative time duration compared to baseline.

#### Echocardiographic parameters

From each echocardiographic report, we collected aortic valve parameters, Simpson biplane left ventricular ejection fraction (LVEF), left ventricular dimensions and mass, diastolic parameters (eg, e′ and E/e′), left atrial volume, right ventricular function, and right ventricular pressure. If biplane LVEF was unavailable, eyeballing LVEF from the baseline echocardiogram was obtained. In addition, we obtained concomitant valvular disease grading from the baseline echocardiogram.

To reduce measurement error of AVA resulting from interobserver variability of left ventricular outflow tract (LVOT) diameter measurement, the median LVOT diameter was calculated for each patient, and the median LVOT diameter was used to calculate AVA using the continuity equation with velocity time integral ratio. If the velocity time integral ratio was unavailable, we determined AVA using the peak jet velocity ratio.

#### Clinical data

We collected the following data from the electronic patient records at the time of baseline echocardiogram: demographic variables, including age, sex, weight and height, medical history, medication use, and routine laboratory values within a 1-year window around baseline. In addition, clinical outcomes, including AVR, all-cause and cardiovascular mortality, hospitalization for heart failure, and myocardial infarction, were recorded until the last known follow-up date.

### Calculation of individual rate of aortic stenosis progression

The annual change in aortic valve parameters (peak jet velocity, AVA, and mean pressure gradient) was computed for each individual patient by fitting an ordinary least squares regression model to the aortic valve parameter measurements over time in years. This yielded an individual regression line for each individual patient, in which the intercept is the fitted baseline severity and the slope of the regression line corresponds to the annual progression rate (Supplemental Figures 1 to 12).

### Statistical analysis

The study cohort was divided into slow and rapid progressors based on the median annual progression rate of AVA, as this is the most important parameter in diagnosing severe aortic stenosis. Baseline characteristics were compared between slow and rapid progressors using 2-sample T tests for continuous variables and Fisher’s exact tests for categorical variables. Continuous variables are presented as the mean ± SD unless stated otherwise. Categorical variables are presented as numbers (percentages).

The association of progression rate with all-cause mortality, cardiovascular mortality, heart failure hospitalization, a composite of heart failure hospitalization and cardiovascular death, and AVR was analyzed using multivariable Cox proportional hazard models, adjusted for the following covariates: age, sex, current smoker, diabetes, hypertension, atrial fibrillation, history of myocardial infarction, history of cerebrovascular accident, obstructive pulmonary disease, peripheral vascular disease, chronic kidney disease, baseline LVEF, and baseline AVA. Proportional hazards assumption was checked using Schoenfeld residuals and showed no violations in all models.

Linear mixed-effect models were constructed to identify variables associated with longitudinal changes in repeated measurements of AVA within the same subject. Mixed-effects models are able to handle correlated data, as is the case for repeated measurements within the same patient, which violate the assumption of independent data points required for ordinary least squares regression. Fixed effects represent population-level effects that do not vary between individuals, whereas random effects account for subject-specific differences that are not explained by the fixed effects. Fixed effects included time in years as a level 1 variable (within-subject level), baseline patient characteristics as a level 2 variable (between-subject level), and the interaction between time and baseline variables. Random effects for each subject were included to allow each subject to have an individual intercept (baseline AVA) and slope (rate of decline). Variables significantly different between slow and rapid progressors in univariable analysis were entered in a multivariable linear mixed-effects model. In addition, the model was adjusted for sex and baseline stroke volume index to obtain a more accurate prediction of AVA.

Using manual backward selection, variables associated with progression rate at *P* < 0.05 significance remained in the final model. The final model was selected using likelihood ratio tests and the Akaike information criterion. Model goodness-of-fit was summarized using marginal R^2^ (indicating the proportion of variance explained by fixed effects only) and conditional R^2^ (indicating variance explained by both fixed and random effects).^8^

All statistical analyses were performed in R version 4.3 (R Core Team, 2023), and 2-sided *P* values <0.05 were considered statistically significant.

## Results

### Baseline characteristics

**Figure 1:**
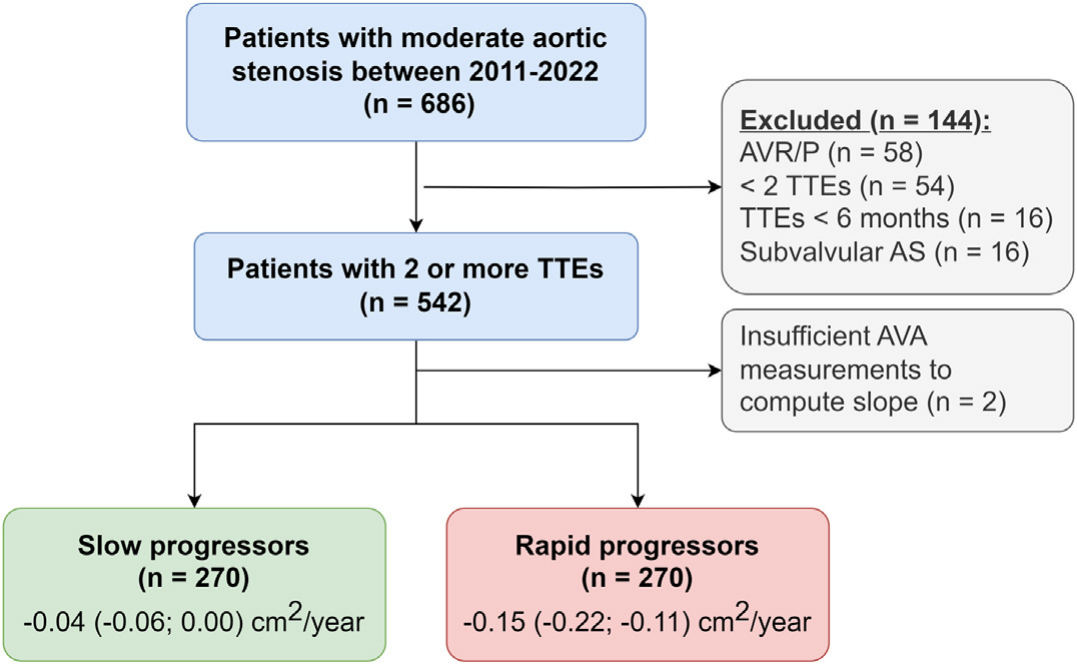
**Study Flow Chart** AS = aortic stenosis; AVA = aortic valve area; AVR/P = aortic valve replacement/valvuloplasty.

We identified 686 patients with moderate aortic stenosis and at least 2 echocardiograms between December 2011 and December 2022, of which 144 patients were excluded due to prior AVR, valve repair, or balloon valvuloplasty before the first exam (n = 58), insufficient exams to determine the progression rate (n = 54), only exams performed within 6 months (n = 16), or subvalvular aortic stenosis or congenital heart disease (n = 16) (Figure 1). Consequently, 540 patients were included in the study with a total number of 2,937 echocardiograms (median number of 5 per patient) and a median follow-up of 3.9 years since baseline (IQR: 2.1-5.9 years). Median age at baseline was 69 years (IQR 59-76), and 62% was male.

### Annual progression rate of aortic valve parameters

In the total cohort, the median decrease in AVA was 0.09 cm^2^/y (IQR: 0.04-0.15 cm^2^/y). Similarly, the median increase in peak jet velocity was 0.17 m/s/y (IQR: 0.09-0.30 m/s/y), and the increase in mean gradient was 3.1 mm Hg/y (IQR: 1.5-5.7 mm Hg/y). The total cohort was divided into slow (median change in AVA −0.04 cm^2^/y) and rapid (−0.15 cm^2^/y) progression of aortic stenosis, indicating progression below or above the median, respectively (Figure 1).

### Baseline differences between slow and rapid progressors

Table 1 shows the patient characteristics of the patients with slow and rapid progression of aortic stenosis. The rapid progression group was older and had a higher proportion of atrial fibrillation, hypertension, and chronic kidney disease compared to patients in the slow progression group. Conversely, the proportion of patients with a bicuspid aortic valve was highest in the slow progression group.

**Table 1:** Baseline Characteristics According to AVA Progression Rate

	Slow (n = 270)	Rapid (n = 270)	Total (N = 540)	*P* Value^a^
Demographics				
Age (y)	61 ± 20	69 ± 10	65 ± 16	<0.001
Male	166 (61.5%)	171 (63.3%)	337 (62.4%)	0.722
Current smoker	79 (29.3%)	91 (33.7%)	170 (31.5%)	0.308
BMI (kg/m^2^)	27.2 (5.6)	28.1 (4.7)	27.7 (5.2)	0.008
Medical history				
Bicuspid aortic valve	66 (24.4%)	25 (9.3%)	91 (16.9%)	<0.001
Diabetes	56 (20.7%)	65 (24.1%)	121 (22.4%)	0.409
Hypertension	144 (53.3%)	179 (66.3%)	323 (59.8%)	0.003
Coronary artery disease	53 (19.6%)	66 (24.4%)	119 (22.0%)	0.213
Myocardial infarction	27 (10.0%)	29 (10.7%)	56 (10.4%)	0.888
Atrial fibrillation	32 (11.9%)	69 (25.6%)	101 (18.7%)	<0.001
Heart failure	19 (7.0%)	21 (7.8%)	40 (7.4%)	0.870
Intracardiac device	11 (4.1%)	18 (6.7%)	29 (5.4%)	0.252
Previous CVA	32 (11.9%)	45 (16.7%)	77 (14.3%)	0.139
Peripheral vascular disease	27 (10.0%)	26 (9.6%)	53 (9.8%)	1.000
Chronic kidney disease	14 (5.2%)	39 (14.4%)	53 (9.8%)	<0.001
Chronic dialysis	7 (2.6%)	25 (9.3%)	32 (5.9%)	0.002
Chest irradiation	11 (4.1%)	17 (6.3%)	28 (5.2%)	0.332
COPD/asthma	46 (17.0%)	36 (13.3%)	82 (15.2%)	0.280
Medication use				
Beta-blocker	105 (38.9%)	125 (46.3%)	230 (42.6%)	0.098
ACE inhibitors	79 (29.3%)	91 (33.7%)	170 (31.5%)	0.308
MRA	6 (2.2%)	17 (6.3%)	23 (4.3%)	0.031
Statin	109 (40.4%)	136 (50.4%)	245 (45.4%)	0.025
Oral anticoagulant	31 (11.5%)	68 (25.2%)	99 (18.3%)	<0.001
Baseline lab values				
Hemoglobin (g/dL)	13.5 ± 1.8	13.4 ± 1.9	13.4 ± 1.8	0.416
Sodium (mmol/L)	140 ± 3	140 ± 3	140 ± 3	0.691
Potassium (mmol/L)	4.2 ± 0.5	4.3 ± 0.6	4.3 ± 0.6	0.057
BUN (mg/dL)	20.4 ± 12.5	24.1 ± 15.6	22.3 ± 14.3	0.006
Creatinine (mg/dL)	1.17 ± 1.21	1.58 ± 2.03	1.38 ± 1.70	0.010
eGFR (CKD-EPI)	75 ± 25	69 ± 26	72 ± 26	0.012
Cholesterol (mg/dL)	177.5 ± 44.6	176.8 ± 50.5	177.2 ± 47.8	0.889
LDL cholesterol (mg/dL)	106.4 ± 39.7	108.3 ± 43.6	107.4 ± 41.8	0.667
HDL cholesterol (mg/dL)	52.8 ± 18.0	49.7 ± 16.0	51.1 ± 17.0	0.085
Triglycerides (mg/dL)	154.5 ± 102.1	168.8 ± 103.0	162.0 ± 102.7	0.223
Baseline echocardiography				
AVA (cm^2^)	1.3 ± 0.3	1.4 ± 0.3	1.3 ± 0.3	<0.001
Peak jet velocity (m/s)	3.1 ± 0.5	3.1 ± 0.4	3.1 ± 0.4	0.297
Mean gradient (mmHg)	22.5 ± 7.0	21.6 ± 5.8	22.0 ± 6.4	0.114
LVEF ≥50%	252 (93.3%)	241 (89.3%)	493 (91.3%)	0.126
SVI ≥35 mL/m^2^	196 (82.0%)	217 (88.6%)	413 (85.3%)	0.053
LVIDd (mm)	46.8 ± 6.8	47.4 ± 7.0	47.1 ± 6.9	0.302
LVISd (mm)	30.2 ± 6.4	31.7 ± 7.4	30.9 ± 6.9	0.018
IVSd (mm)	11.1 ± 2.4	11.5 ± 2.3	11.3 ± 2.4	0.050
PWd (mm)	9.7 ± 1.8)	10.3 ± 2.3	10.0 ± 2.1	0.004
LAVI (ml/m^2^)	33.0 ± 11.6	40.3 ± 20.3	36.6 ± 16.9	<0.001
RWT	0.43 ± 0.10	0.44 ± 0.14	0.43 ± 0.12	0.129
LVMI (g/m^2^)	89.2 ± 25.7	97.7 ± 29.3	93.4 ± 27.8	<0.001
e’ average (m/s)	9.2 ± 3.3	7.8 ± 2.0	8.5 ± 2.8	<0.001
Mitral regurgitation				0.004
None-Trace	216 (80.0%)	189 (70.5%)	405 (75.3%)	
Mild	43 (15.9%)	59 (22.0%)	102 (19.0%)	
Moderate	8 (3.0%)	20 (7.5%)	28 (5.2%)	
Severe	3 (1.1%)	0 (0.0%)	3 (0.6%)	
Aortic regurgitation				0.151
None-Trace	165 (61.3%)	186 (69.4%)	351 (65.4%)	
Mild	74 (27.5%)	64 (23.9%)	138 (25.7%)	
Moderate	27 (10.0%)	17 (6.3%)	44 (8.2%)	
Severe	3 (1.1%)	1 (0.4%)	4 (0.7%)	
Tricuspid regurgitation				0.195
None-Trace	210 (78.7%)	195 (73.3%)	405 (76.0%)	
Mild	49 (18.4%)	53 (19.9%)	102 (19.1%)	
Moderate	7 (2.6%)	14 (5.3%)	21 (3.9%)	
Severe	1 (0.4%)	4 (1.5%)	5 (0.9%)	

Values are mean ± SD or n (%).

ACE = angiotensin-converting enzyme; AVA = aortic valve area; BMI = body mass index; BUN = blood urea nitrogen; COPD = chronic obstructive pulmonary disease; CVA = cerebrovascular accident; eGFR = estimated glomerular filtration rate; HDL = high-density lipoprotein; IVSd = interventricular septum diameter; LAVI = left atrial volume index; LDL = low-density lipoprotein; LVEF = left ventricular ejection fraction; LVIDd = left ventricular internal end-diastolic diameter; LVISd = left ventricular internal end-systolic diameter; LVMI = left ventricular mass index; MRA = mineralocorticoid receptor antagonist; PWd = posterior wall diameter; RWT = relative wall thickness; SVI = stroke volume index.

a *P* values depict comparisons between slow and rapid progressors using 2-sample T-tests for continuous variables and Fisher’s exact tests for categorical variables.

Baseline blood urea nitrogen was higher in the rapid progression groups compared to the slow progression group. Baseline lipid levels were similar between slow and rapid progressors. In addition, the baseline left atrial volume index and left ventricular mass index were higher in the rapid progression group compared to the slow progression group.

### Association of progression rate with clinical outcomes

**Figure 2:**
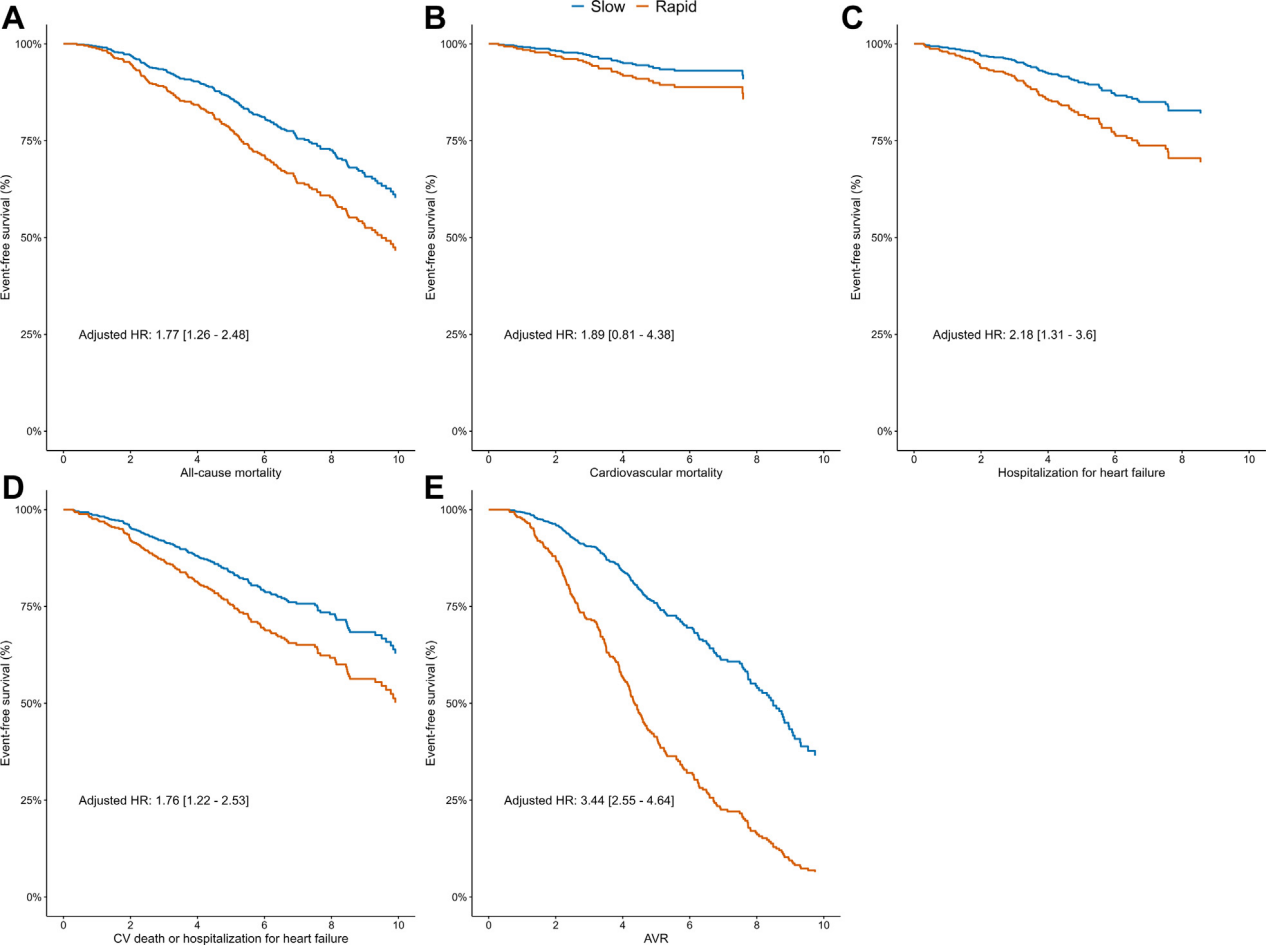
**Association of Rate of Aortic Stenosis Progression With Long-Term Clinical Outcomes Up to 10 Years** (A) All-cause mortality; (B) cardiovascular mortality; (C) heart failure hospitalization; (D) composite of heart failure hospitalization and cardiovascular death; and (E) aortic valve replacement. Curves depict event-free survival for slow and rapid progression of aortic stenosis adjusted in multivariable analysis, and time is presented in years. AVR = aortic valve replacement; CV death = cardiovascular death; HR = hazard ratio.

Table 2 shows clinical outcomes in patients with slow and rapid progression. Patients in the rapid progression group had a higher rate of AVR (55 vs 41%; *P* < 0.001), death (41 vs 26%; *P* < 0.001), and hospitalization for heart failure (21 vs 11%; *P* = 0.002) compared to the slow progression group. Kaplan-Meier curves depicted in Figure 2 show a higher rate of all-cause mortality, heart failure hospitalization, composite of heart failure hospitalization and cardiovascular death, and AVR in patients with a more rapid progression of aortic stenosis, adjusted for baseline confounders. In multivariable Cox regression adjusted for age, sex, smoking, diabetes, atrial fibrillation, myocardial infarction, hypertension, stroke, pulmonary disease, peripheral artery disease, chronic kidney disease, LVEF, and baseline AVA, rapid progression was independently associated with all-cause mortality (HR: 1.77, 95% CI: 1.26-2.48; Supplemental Table 1) and AVR (HR: 3.44, 95% CI: 2.55-4.64).

**Table 2:** Clinical Outcomes According to AVA Rate of Progression

	Slow (n = 270)	Rapid (n = 270)	Total (N = 540)	*P* Value^a^
FU duration since baseline (y)	5.1 ± 2.8	3.4 ± 2.0	4.3 ± 2.6	<0.001
AVR	111 (41.1%)	148 (54.8%)	259 (48.0%)	0.002
Aortic valve replacement				0.004
No	159 (58.9%)	122 (45.2%)	281 (52.0%)	
SAVR	71 (26.3%)	87 (32.2%)	158 (29.3%)	
TAVR	40 (14.8%)	61 (22.6%)	101 (18.7%)	
Death	69 (25.6%)	110 (40.7%)	179 (33.1%)	<0.001
Hospitalization for heart failure	29 (10.8%)	56 (20.9%)	85 (15.9%)	0.002
New-onset heart failure	26 (10.4%)	35 (14.2%)	61 (12.3%)	0.220
New-onset atrial fibrillation	48 (20.1%)	51 (24.6%)	99 (22.2%)	0.256
Myocardial infarction				0.810
No	252 (93.3%)	249 (92.2%)	501 (92.8%)	
NSTEMI	13 (4.8%)	14 (5.2%)	27 (5.0%)	
STEMI	5 (1.9%)	7 (2.6%)	12 (2.2%)	
Percutaneous coronary intervention	26 (9.6%)	30 (11.1%)	56 (10.4%)	0.672
CABG	17 (6.3%)	21 (7.8%)	38 (7.0%)	0.614
Cerebrovascular accident	14 (5.2%)	20 (7.5%)	34 (6.3%)	0.376
Peripheral artery disease intervention/surgery	10 (4.0%)	16 (6.2%)	26 (5.2%)	0.316
Device implantation				0.883
No	205 (92.3%)	208 (90.4%)	413 (91.4%)	
Pacemaker	12 (5.4%)	16 (7.0%)	28 (6.2%)	
ICD	1 (0.5%)	2 (0.9%)	3 (0.7%)	

Values are mean ± SD or n (%).

AVR = aortic valve replacement; CABG = coronary artery bypass graft; FU = follow-up; ICD = implantable cardioverter defibrillator; NSTEMI = non-ST-segment elevation myocardial infarction; SAVR = surgical aortic valve replacement; STEMI = ST-segment elevation myocardial infarction; TAVR = transcatheter aortic valve replacement.

a *P* values depict comparisons between slow and rapid progressors using 2-sample t-tests for continuous variables and Fisher’s exact tests for categorical variables.

### Factors associated with a change in aortic valve area over time

Table 3 shows the final linear mixed-effects model with AVA as the outcome variable, which calculates the predicted AVA for subject *i* in *x* years from baseline. The intercept represents the baseline AVA at the first echocardiographic diagnosis of aortic stenosis. On average, baseline AVA was increased in males (0.105 cm^2^) with a history of chronic kidney disease (0.123 cm^2^) and a higher stroke volume index (0.085 cm^2^ per 10 mL/m^2^ increase in stroke volume index).

**Table 3:** Model Summary of Linear Mixed-Effects Prediction Model With AVA as Outcome, to Compute Predicted AVA in Years From Baseline

	Estimates	95% CI	*P* Value
Intercept	0.877	0.773-0.981	<0.001
Male sex	0.105	0.060-0.149	<0.001
Chronic kidney disease (eGFR <60)	0.123	0.043-0.204	<0.001
Baseline SVI (per 10 mL/m^2^)	0.085	0.064-0.107	<0.001
Time (y)	0.082	0.045-0.120	<0.001
Time × age (per 10 y)	−0.013	−0.016 to −0.010	<0.001
Time × atrial fibrillation	−0.021	−0.038 to −0.004	0.014
Time × chronic kidney disease	−0.059	−0.083 to −0.034	<0.001
Time × baseline LVMI (per 50 g/m^2^)	−0.019	−0.031 to −0.007	0.002
Time × baseline SVI (per 10 mL/m^2^)	−0.008	−0.014 to −0.002	0.009
N	449		
Observations	2332		
Marginal R^2^/Conditional R^2^	0.342/0.736		

AVA = aortic valve area; eGFR = estimated glomerular filtration rate; LVMI = left ventricular mass index; SVI = stroke volume index.

The interaction terms of time with predictors depicted in Table 3 represent the effect of each predictor on progression rate. Predictors associated with a faster decline in AVA were older age (−0.013 cm^2^/y for every 10 year increase in age), atrial fibrillation (−0.021 cm^2^/y), chronic kidney disease (−0.059 cm^2^/y), a higher baseline left ventricular mass index (−0.019 cm^2^/y for every 50 g/m^2^ increase), and a higher baseline stroke volume index (−0.008 cm^2^/y for every 10 mL/m^2^ increase). A modest negative correlation existed between intercept and slope (rho = −0.22), indicating that a higher intercept (baseline AVA) was on average associated with a slower progression rate. The marginal R^2^ of the final model was 0.34, and the conditional R^2^ was 0.74. The model fit measures and random effects for different models are available in Supplemental Table 2. The calculation and predictions of the final model are summarized in the Central Illustration and the predicted trajectories of the patients in our sample based on the model are shown in Supplemental Figures 1 to 12.

**Central Illustration:**
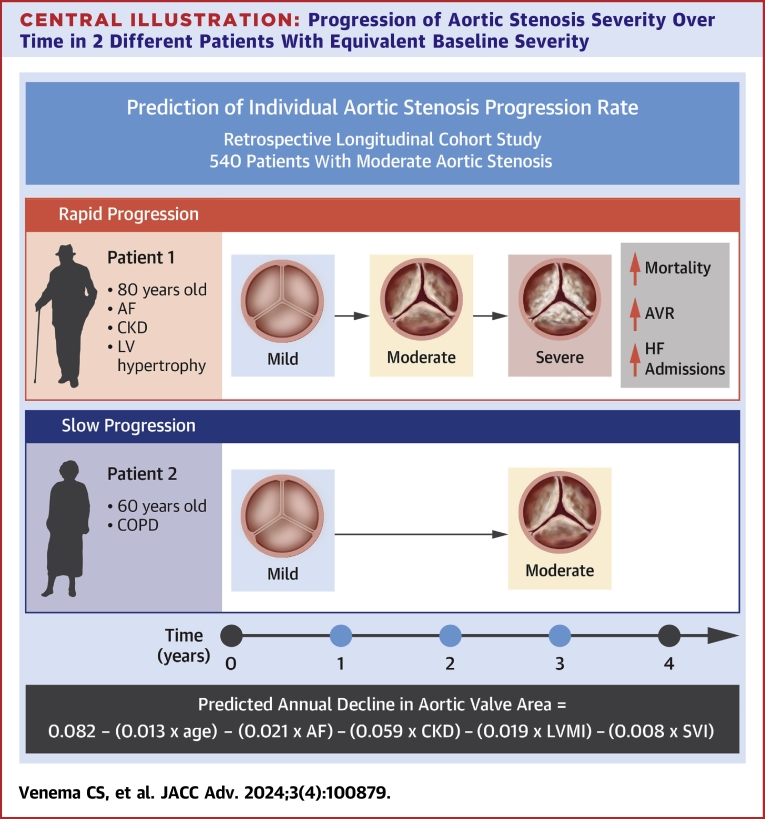
**Progression of Aortic Stenosis Severity Over Time in 2 Different Patients With Equivalent Baseline Severity** Patient 1 has rapid progression and is an elderly individual of 80 years old with a history of atrial fibrillation and chronic kidney disease, and has left ventricular hypertrophy (elevated LVMI). We found that rapid progression is associated with increased mortality, aortic valve replacement, and heart failure admissions. Patient 2 is 60 years old with a history of COPD and has slow progression of aortic stenosis. The equation at the bottom of the figure depicts the model calculation to predict the annual decline in aortic valve area, with age per 10-year increase, LVMI per 50 g/m^2^ increase, and SVI per 10 mL/m^2^ increase. AF = atrial fibrillation; AVA = aortic valve area; AVR = aortic valve replacement; CKD = chronic kidney disease (estimated glomerular filtration rate <60 ml/min/1.73 m^2^); COPD = chronic obstructive pulmonary disease; HF = heart failure; LVMI = left ventricular mass index; SVI = stroke volume index.

## Discussion

The main findings of this longitudinal, retrospective, single-center cohort study analyzing individual patterns of progression of aortic stenosis were as follows: 1) the average annual decline in AVA was 0.09 cm^2^, and the annual increase in peak jet velocity and mean gradient were 0.17 m/s and 3.1 mmHg, respectively; 2) the progression of aortic stenosis over time was linear in the majority of patients; 3) in linear mixed-effects analysis, older age, atrial fibrillation, chronic kidney disease, and left ventricular mass index were associated with a faster decline in AVA; and 4) faster progression was independently associated with all-cause mortality and AVR.

### Rate of aortic stenosis progression

The average rate of aortic stenosis progression in our cohort was similar to previous reports, with a previously reported decline in AVA ranging between 0.03 and 0.14 cm^2^ per year and an increase in peak jet velocity ranging between 0.15 and 0.40 m/s per year.^5^^,^^9^, ^10^, ^11^, ^12^, ^13^, ^14^, ^15^, ^16^, ^17^, ^18^, ^19^, ^20^, ^21^, ^22^ Several studies used the difference between the first and last available echocardiogram or the time to severe aortic stenosis to determine the progression rate.^17^^,^^22^ In our study, we used all available echocardiographic measurements of each patient to obtain a subject-specific slope. This method provides an accurate assessment of the progression rate, with the caveat that the progression rate may be underestimated or overestimated in the case of only 2 measurements. We assumed a linear rate of progression, which was supported by our finding that in most patients, aortic valve parameters followed a straight line over time. Some studies suggest that the progression of aortic stenosis includes a propagation phase in which valve calcification becomes self-perpetuating.^23^^,^^24^ This phenomenon of accelerated progression has been observed in a study investigating progression to severe aortic stenosis, in which progression was accelerated at an AVA of 1.2 cm^2^.^21^ A similar finding was observed in another study of patients with bicuspid vs tricuspid aortic stenosis, in which progression accelerated at a mean gradient of 30 mm Hg or peak jet velocity of 3.5 m/s.^19^ However, aortic valve parameters over time in different subjects were pooled together in these studies. This does not take into account the within-subject correlation between serial measurements. Lastly, a linear model was a good fit for measurements of individual subjects in our study and is easier to interpret.

### Association of rate of progression with clinical outcomes

We confirmed that the rapid progression of aortic stenosis is independently associated with all-cause mortality and AVR, in line with previous reports.^5^^,^^22^^,^^25^ We found a higher rate of heart failure hospitalizations in patients with rapid progression. It can be hypothesized that a failing left ventricle cannot compensate for more rapid increases in afterload due to progressive aortic stenosis, leading to a higher risk of heart failure hospitalization. Patients with a high risk of rapid progression should therefore be regularly monitored. Future studies need to investigate whether patients at high risk for rapid progression may benefit from an earlier timing of intervention, which becomes increasingly relevant with the EXPAND TAVR II (Evolut™ EXPAND TAVR II Pivotal Trial) (NCT05149755) and PROGRESS (Evolut™ EXPAND TAVR II Pivotal Trial) (NCT04889872) trials now investigating the benefit of early intervention in patients with symptomatic moderate aortic stenosis.

### Factors associated with rate of aortic stenosis progression

A recent meta-analysis showed that a higher baseline severity of aortic stenosis is associated with a faster progression rate. In contrast, we found a modest negative correlation between intercept and slope, indicating that higher baseline severity is associated with a lower rate of progression.^6^ It should be noted that the overall heterogeneity between studies in this meta-analysis was substantial, and insufficient data were available to adjust for potential confounders such as age and comorbidities. Also, methodological differences may play a role. For instance, when the time to severe aortic stenosis is used to describe the progression rate, it is apparent that higher baseline aortic stenosis severity reduces the time to reach severe aortic stenosis.^22^ Besides, we specifically included patients with at least one echocardiogram meeting moderate aortic stenosis criteria. Subsequently, all patients with mild aortic stenosis at baseline reached moderate aortic stenosis in our study. This may have contributed to the finding that lower baseline severity was associated with faster progression in our study.

We identified several clinical factors that were associated with more rapid progression, and previous reports can confirm the validity of the predictors identified in our model. Similar to our findings, a cohort study including patients with moderate aortic stenosis found that higher baseline age, atrial fibrillation, low estimated glomerular filtration rate, dyslipidemia, and higher posterior wall thickness were associated with progression to severe aortic stenosis.^22^ Higher age is associated with higher degrees of valvular calcification, and it is known that the prevalence of aortic stenosis is higher in elderly patients, which likely explains the association of age with faster progression.^26^ Higher baseline stroke volume index was associated with a more rapid decline in AVA, suggesting that patients with low-flow aortic stenosis have a more gradual decline in AVA compared to those with high flow. Chronic kidney disease and albuminuria have been identified as major risk factors for rapid progression of aortic stenosis.^22^^,^^27^^,^^28^ Multiple pathophysiological processes in chronic kidney disease have been implicated in the progression of aortic valve calcification. In particular, metabolic disturbances in calcium and phosphate homeostasis, fluid overload inducing increased shear stress, and uremic toxins are involved, particularly in dialysis patients.^26^^,^^27^ The higher left ventricular mass index was associated with a faster progression rate in our study, which aligns with previous findings.^11^ It can be hypothesized that patients with faster progression have more restricted leaflet movement leading to higher pressure gradients and increased wall stress during periods with increased flow, resulting in a greater degree of left ventricular remodeling and thus higher left ventricular mass. This hypothesis can be further extended to the association of atrial fibrillation with the faster progression rate found in this study, as left atrial dilatation and the development of atrial fibrillation are essential sequelae of aortic stenosis.^29^ In addition, using vitamin K antagonists in patients with atrial fibrillation may contribute to valve calcification and faster progression and has also been associated with worse outcomes after transcatheter aortic valve implantation.^30^, ^31^, ^32^

To our knowledge, this is the first study that applies a subject-specific approach to predict the progression rate of aortic stenosis. Using a mixed-effects modeling approach, we could use all available data and accurately model individual longitudinal changes in aortic stenosis progression.

### Study limitations

Our study has several limitations. This was a single-center study including patients from a tertiary referral center, which may not be representative of patients in primary and secondary centers. Also, the retrospective design depends on the accuracy and completeness of the documentation in medical charts, which may have resulted in missing information. To reduce heterogeneity in the study cohort and inaccuracies and variability in echocardiographic measurements, we selected subjects with at least one echocardiogram meeting the criteria for moderate aortic stenosis and used only echocardiograms after December 2011. Using an extensive search query in the hospital's electronic records, we identified all patients with these criteria. We were thus able to minimize selection bias. Nonetheless, possible improvements in measurement techniques and technological advances during the study period could have influenced the accuracy of the measurements over time.

## Conclusions

We showed that the progression of aortic stenosis follows an individual and linear pattern. Faster progression of aortic stenosis was associated with higher age, atrial fibrillation, chronic kidney disease, and left ventricular mass index and was associated with worse outcomes. These findings indicate that aortic stenosis patients with these characteristics may require closer monitoring. Our model provides a step toward the development of a patient-specific aortic stenosis progression rate calculator to identify patients at risk for rapid progression at baseline and to tailor follow-up intervals, which becomes increasingly important with the rising worldwide burden of valvular heart disease.^2^

## Funding support and author disclosures

The authors have reported that they have no relationships relevant to the contents of this paper to disclose.

PERSPECTIVES **COMPETENCY IN MEDICAL KNOWLEDGE:** Aortic stenosis progression rate is heterogeneous and follows a linear trajectory in the majority of patients. Rapid progression is independently associated with all-cause mortality and aortic valve replacement. Older age, greater left ventricular mass index, atrial fibrillation, and chronic kidney disease at the baseline diagnosis of aortic stenosis are associated with a faster decline in the aortic valve area.

**TRANSLATIONAL OUTLOOK:** Our model provides a step toward the development of a patient-specific aortic stenosis progression rate calculator to identify patients at risk for rapid progression at baseline and to tailor follow-up intervals, which becomes increasingly important with the rising worldwide burden of valvular heart disease. Furthermore, our study could help identify patients for future trials investigating therapies to delay aortic stenosis progression.
