# Supplementary material for: Prediction of the Individual Aortic Stenosis Progression Rate and its Association With Clinical Outcomes
Source: JACC Adv. 2024 Mar 6;3(4):100879. doi: 10.1016/j.jacadv.2024.100879 (PMC11198185; doi:10.1016/j.jacadv.2024.100879)
Supplement: Supplemental Figures and Tables [file mmc1.pdf]

Supplementary data

- **Supplementary table 1.** Multivariable Cox proportional hazard models with all-cause mortality, cardiovascular mortality, heart failure hospitalization, a composite of cardiovascular mortality and heart failure hospitalization, and aortic valve replacement as outcomes. AVA = aortic valve area; CI: confidence interval; CVA: cerebrovascular accident; HR: hazard ratio; LVEF: left ventricular ejection fraction.
- **Supplementary table 2.** Model summaries of different linear mixed effects model with aortic valve area as outcome. The unconditional means model estimates the average AVA at any time point, whereas the unconditional growth model estimates the baseline AVA (the intercept) and the average rate of decline per year without including any covariates.
- **Supplementary figures 1-12.** Progression of aortic valve area (AVA) over time in all individual subjects. Serial AVA measurements were plotted over time and an ordinary least squares regression line was fitted to each plot, resulting in an individual intercept and slope for each patient (blue line). The red line depicts the predicted trajectory of each patient according to the linear mixed effects model. The black dotted vertical line indicates the baseline diagnosis of aortic stenosis (first TTE with peak jet velocity ≥ 2.6 m/s), and the red dotted vertical line indicates timing of aortic valve replacement. Background colours indicated no/mild, moderate, and severe aortic stenosis from top to bottom, respectively, based on aortic valve area. AVA: aortic valve area.

Supplementary table 1. Multivariable Cox proportional hazard models with all-cause mortality, cardiovascular mortality, heart failure hospitalization, a composite of cardiovascular mortality and heart failure hospitalization, and aortic valve replacement as outcomes. AVA = aortic valve area; CI: confidence interval; CVA: cerebrovascular accident; HR: hazard ratio; LVEF: left ventricular ejection fraction.

	**All-cause mortality**			**Cardiovascular mortality**			**Heart failure hospitalization**			**Composite of cardiovascular mortality and heart failure hospitalization**			**AVR**		
Predictors	***HR***	***95% CI***	***p***	***HR***	***95% CI***	***p***	***HR***	***95% CI***	***p***	***HR***	***95% CI***	***p***	***HR***	***95% CI***	***p***
**Rapid AS progression**	1.77	1.26 – 2.48	**0.001**	1.89	0.81 – 4.38	0.140	2.18	1.31 – 3.60	**0.003**	1.76	1.22 – 2.53	**0.002**	3.44	2.55 – 4.64	**<0.001**
**Age at diagnosis**	1.07	1.05 – 1.08	**<0.001**	1.14	1.08 – 1.20	**<0.001**	1.06	1.03 – 1.09	**<0.001**	1.07	1.05 – 1.09	**<0.001**	0.99	0.99 – 1.00	0.271
**Male sex**	1.29	0.93 – 1.79	0.133	1.03	0.47 – 2.27	0.937	1.15	0.71 – 1.86	0.582	1.06	0.74 – 1.52	0.744	1.39	1.06 – 1.82	**0.019**
**Current smoker**	0.98	0.71 – 1.35	0.883	1.39	0.64 – 3.01	0.404	1.14	0.71 – 1.83	0.582	1.00	0.71 – 1.43	0.984	1.28	0.97 – 1.69	0.077
**Diabetes**	1.48	1.05 – 2.09	**0.025**	3.34	1.48 – 7.55	**0.004**	1.55	0.94 – 2.53	0.083	1.49	1.03 – 2.16	**0.036**	1.30	0.94 – 1.79	0.117
**Atrial fibrillation**	1.13	0.79 – 1.61	0.517	1.32	0.55 – 3.21	0.534	1.50	0.91 – 2.47	0.110	1.67	1.16 – 2.40	**0.006**	1.42	1.01 – 2.00	**0.041**
**Previous myocardial infarction**	1.15	0.73 – 1.81	0.535	1.78	0.57 – 5.58	0.322	0.94	0.49 – 1.82	0.860	1.37	0.87 – 2.16	0.177	0.88	0.56 – 1.39	0.593
**Hypertension**	0.90	0.64 – 1.25	0.522	0.42	0.19 – 0.90	**0.026**	0.65	0.41 – 1.05	0.082	0.76	0.53 – 1.10	0.144	1.09	0.82 – 1.47	0.546
**Previous CVA**	1.38	0.96 – 1.98	0.086	1.71	0.76 – 3.86	0.192	1.18	0.67 – 2.08	0.561	1.37	0.92 – 2.03	0.122	1.00	0.67 – 1.48	0.991
**COPD/asthma**	1.28	0.86 – 1.90	0.216	0.81	0.30 – 2.20	0.682	1.75	1.00 – 3.03	**0.048**	1.37	0.87 – 2.14	0.172	1.20	0.80 – 1.78	0.377

**Peripheral vascular disease**	1.85	1.23 – 2.77	**0.003**	3.88	1.64 – 9.16	**0.002**	2.70	1.50 – 4.86	**0.001**	2.14	1.38 – 3.32	**0.001**	0.78	0.48 – 1.26	0.311
**Chronic kidney disease (eGFR<60)**	2.55	1.64 – 3.96	**<0.001**	2.40	0.83 – 6.94	0.105	2.25	1.18 – 4.29	**0.014**	1.94	1.17 – 3.23	**0.011**	0.68	0.42 – 1.10	0.118
**LVEF**	0.97	0.95 – 0.99	**0.008**	0.95	0.90 – 0.99	**0.016**	0.93	0.90 – 0.95	**<0.001**	0.94	0.92 – 0.97	**<0.001**	1.01	0.99 – 1.03	0.442
**Baseline AVA**	0.23	0.11 – 0.46	**<0.001**	0.20	0.03 – 1.23	0.082	0.44	0.16 – 1.16	0.096	0.34	0.16 – 0.70	**0.004**	0.21	0.12 – 0.34	**<0.001**
**Observations**	518			454			516			518			520		
**R^2 Nagelkerke**	0.291			0.305			0.235			0.306			0.184		

Supplementary table 2. Model summaries of different linear mixed effects model with aortic valve area as outcome. The unconditional means model estimates the average AVA at any time point, whereas the unconditional growth model estimates the baseline AVA (the intercept) and the average rate of decline per year without including any covariates.

	**Unconditional means model**		**Unconditional growth model**		**Model 1**		**Model 2**		**Final model**	
Predictors	*Estimates*	*p*	*Estimates*	*p*	*Estimates*	*p*	*Estimates*	*p*	*Estimates*	*p*
(Intercept)	1.19	**<0.001**	1.34	**<0.001**	1.05	**<0.001**	1.23	**<0.001**	0.88	**<0.001**
Time compared to baseline (years)			-0.08	**<0.001**	0.05	0.334	0.05	**0.001**	0.08	**<0.001**
Age at diagnosis					-0.00	0.334				
BMI (kg/m^2)					0.00	0.145				
Bicuspid aortic valve					-0.01	0.781				
Hypertension: Yes					-0.01	0.788				
Atrial fibrillation: Yes					-0.02	0.587				
Chronic kidney disease (eGFR<60)					0.13	**0.003**	0.12	**0.003**	0.12	**0.003**
Male sex									0.10	**<0.001**
Stroke Volume Index									0.09	**<0.001**
LVEF					0.00	0.300				
LVMI					0.00	**0.006**	0.00	**0.012**		
Time * age					-0.00	**<0.001**	-0.00	**<0.001**		
Time * BMI					-0.00	0.773				
Time * bicuspid aortic valve					0.01	0.184				

Time * hypertension			0.01	0.061				
Time * atrial fibrillation			-0.02	**0.013**	-0.02	**0.003**	-0.02	**0.014**
Time * chronic kidney disease			-0.05	**<0.001**	-0.05	**<0.001**	-0.06	**<0.001**
Time * LVEF			-0.00	0.832				
Time * LVMI			-0.00	**<0.001**	-0.00	**0.001**		
Time * age (per 10 years)							-0.01	**<0.001**
Time * LVMI (per 50 g/m^2)							-0.02	**0.002**
Time * SVI (per 10 mL/m^2)							-0.01	**0.009**
**Random Effects**								
σ^2	0.077	0.039	0.040		0.040		0.039	
τ_{00}	0.037 $_{id}$	0.058 $_{id}$	0.053 $_{id}$		0.054 $_{id}$		0.044 $_{id}$	
τ_{11}		0.003 $_{id.time}$	0.002 $_{id.time}$		0.002 $_{id.time}$		0.002 $_{id.time}$	
ρ_{01}		-0.363 $_{id}$	-0.318 $_{id}$		-0.316 $_{id}$		-0.218 $_{id}$	
ICC	0.324	0.656	0.602		0.606		0.599	
N	542 $_{id}$	542 $_{id}$	495 $_{id}$		495 $_{id}$		449 $_{id}$	
Observations	2785	2785	2580		2580		2332	
Marginal R^2 / Conditional R^2	0.000 / 0.324	0.257 / 0.745	0.297 / 0.720		0.292 / 0.721		0.342 / 0.736	
AIC	1434.570	355.778	425.880		326.254		217.254	

AIC: Akaike information criterion; eGFR: estimated glomerular filtration rate; LVMI: left ventricular mass index; SVI: stroke volume index; σ^2: within-subject (level 1) residual variance; $\tau_{00\ id}$: between-subject residual variance in intercept; $\tau_{11\ id.time}$: between-subject residual variance in slope; $\rho_{01\ id}$: intercept-slope correlation.

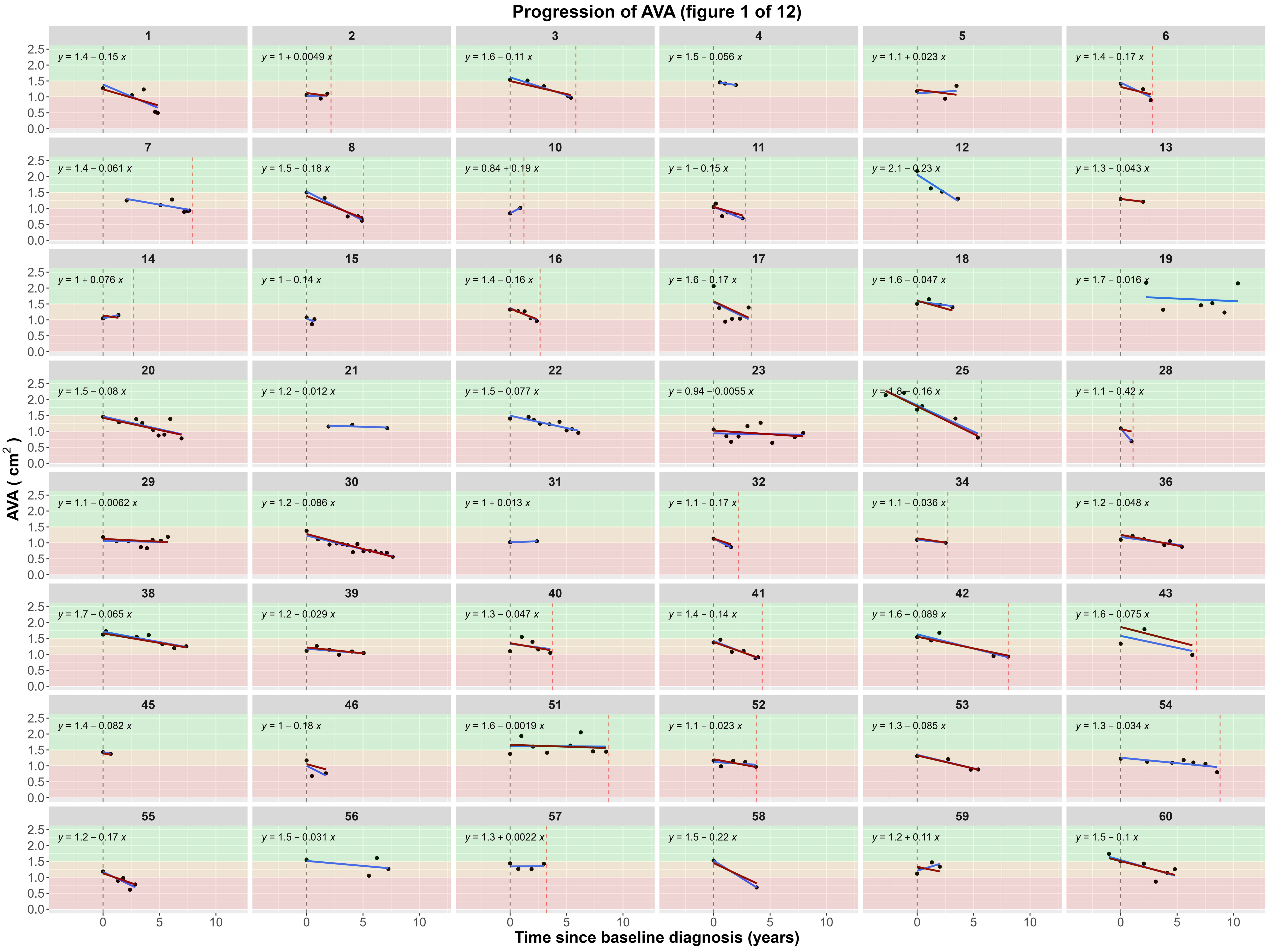
Progression of AVA (figure 1 of 12)
AVA (cm2)
Time since baseline diagnosis (years)
0.0 0.5 1.0 1.5 2.0 2.5
0 5 10
1
y = 1.4 − 0.15 x
2
y = 1 + 0.0049 x
3
y = 1.6 − 0.11 x
4
y = 1.5 − 0.056 x
5
y = 1.1 + 0.023 x
6
y = 1.4 − 0.17 x
7
y = 1.4 − 0.061 x
8
y = 1.5 − 0.18 x
10
y = 0.84 + 0.19 x
11
y = 1 − 0.15 x
12
y = 2.1 − 0.23 x
13
y = 1.3 − 0.043 x
14
y = 1 + 0.076 x
15
y = 1 − 0.14 x
16
y = 1.4 − 0.16 x
17
y = 1.6 − 0.17 x
18
y = 1.6 − 0.047 x
19
y = 1.7 − 0.016 x
20
y = 1.5 − 0.08 x
21
y = 1.2 − 0.012 x
22
y = 1.5 − 0.077 x
23
y = 0.94 − 0.0055 x
25
y = 1.8 − 0.16 x
28
y = 1.1 − 0.42 x
29
y = 1.1 − 0.0062 x
30
y = 1.2 − 0.086 x
31
y = 1 + 0.013 x
32
y = 1.1 − 0.17 x
34
y = 1.1 − 0.036 x
36
y = 1.2 − 0.048 x
38
y = 1.7 − 0.065 x
39
y = 1.2 − 0.029 x
40
y = 1.3 − 0.047 x
41
y = 1.4 − 0.14 x
42
y = 1.6 − 0.089 x
43
y = 1.6 − 0.075 x
45
y = 1.4 − 0.082 x
46
y = 1 − 0.18 x
51
y = 1.6 − 0.0019 x
52
y = 1.1 − 0.023 x
53
y = 1.3 − 0.085 x
54
y = 1.3 − 0.034 x
55
y = 1.2 − 0.17 x
56
y = 1.5 − 0.031 x
57
y = 1.3 + 0.0022 x
58
y = 1.5 − 0.22 x
59
y = 1.2 + 0.11 x
60
y = 1.5 − 0.1 x

Progression of AVA (figure 2 of 12)

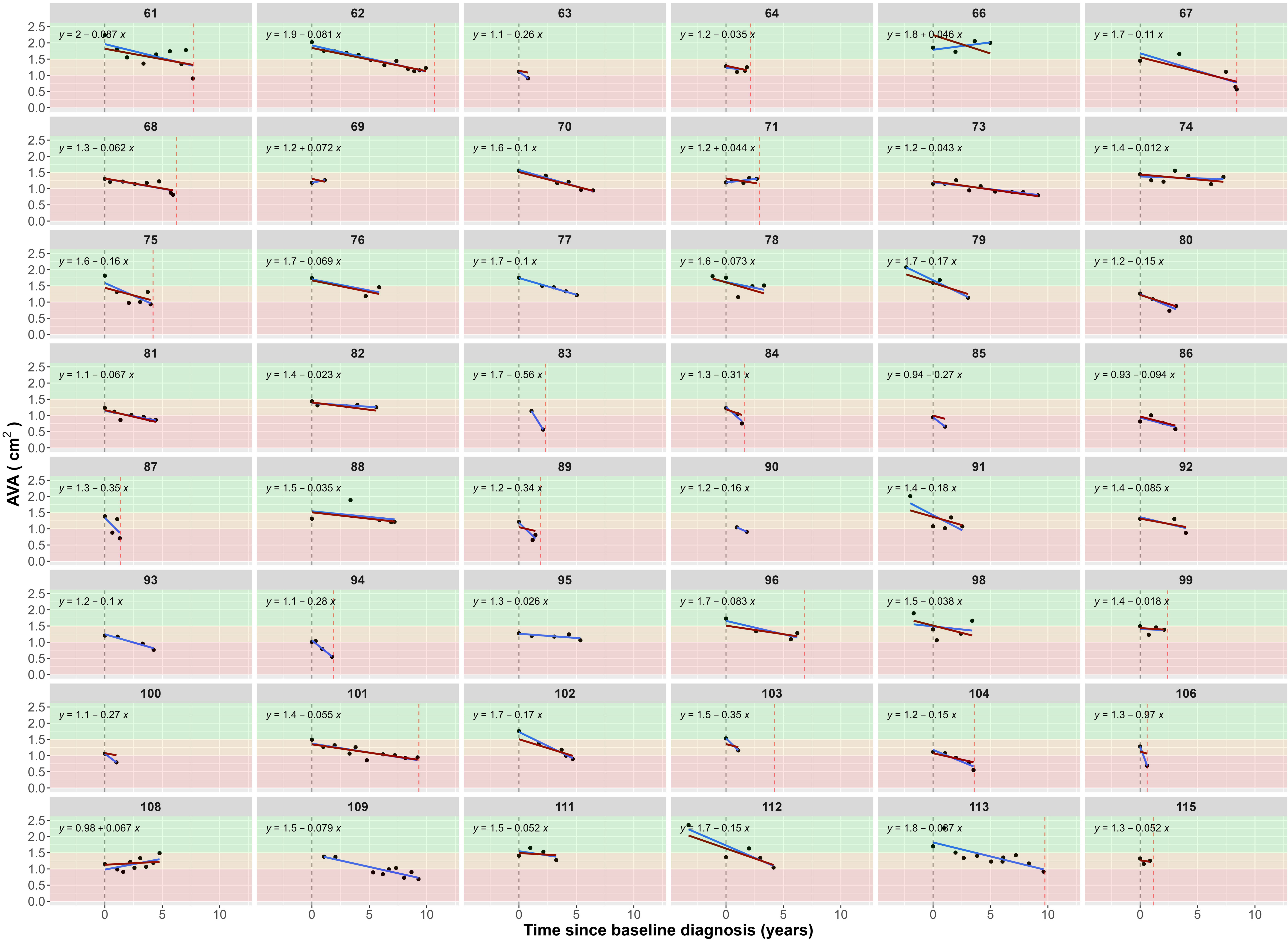

Progression of AVA (figure 3 of 12)

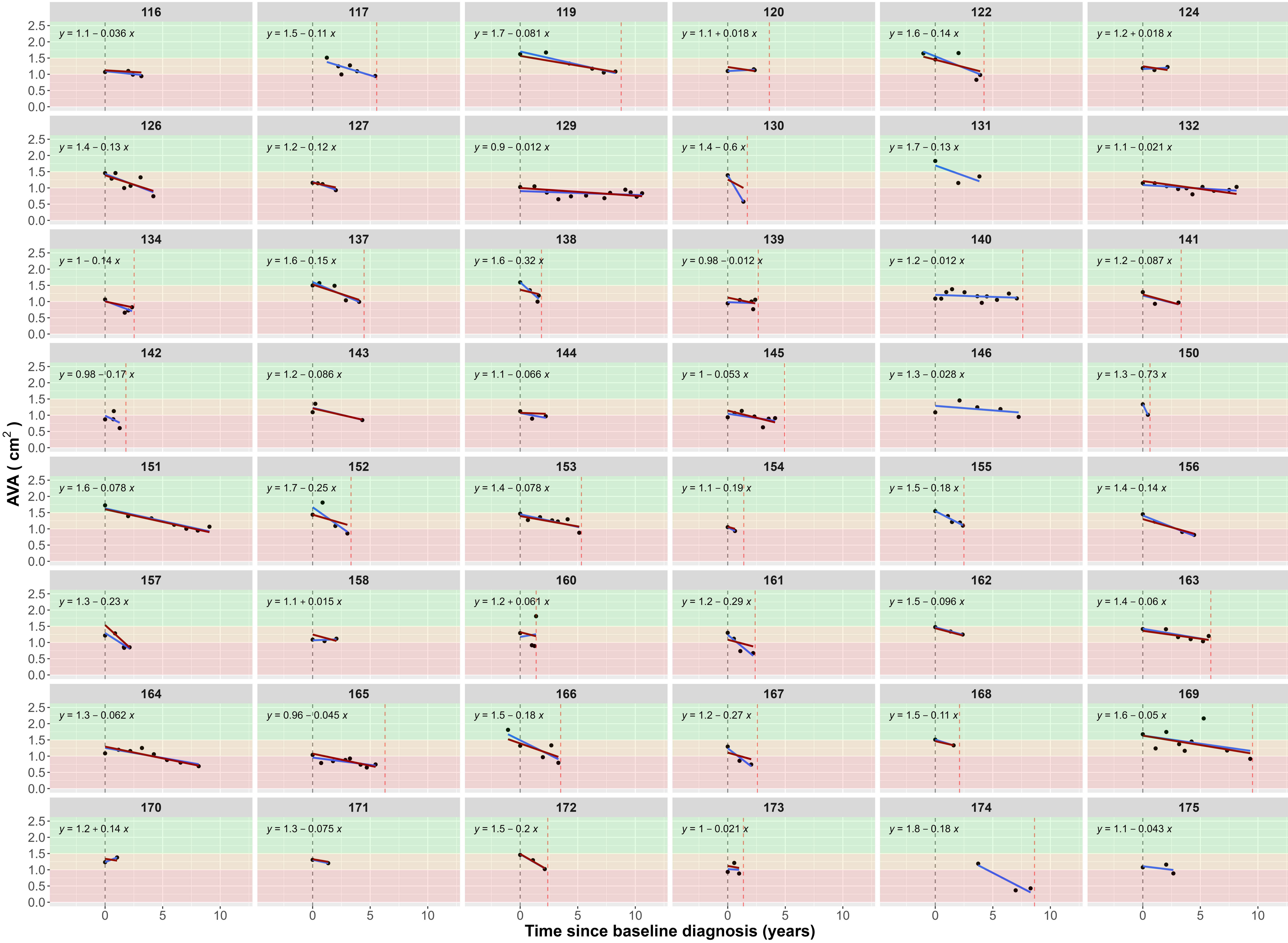

Progression of AVA (figure 4 of 12)

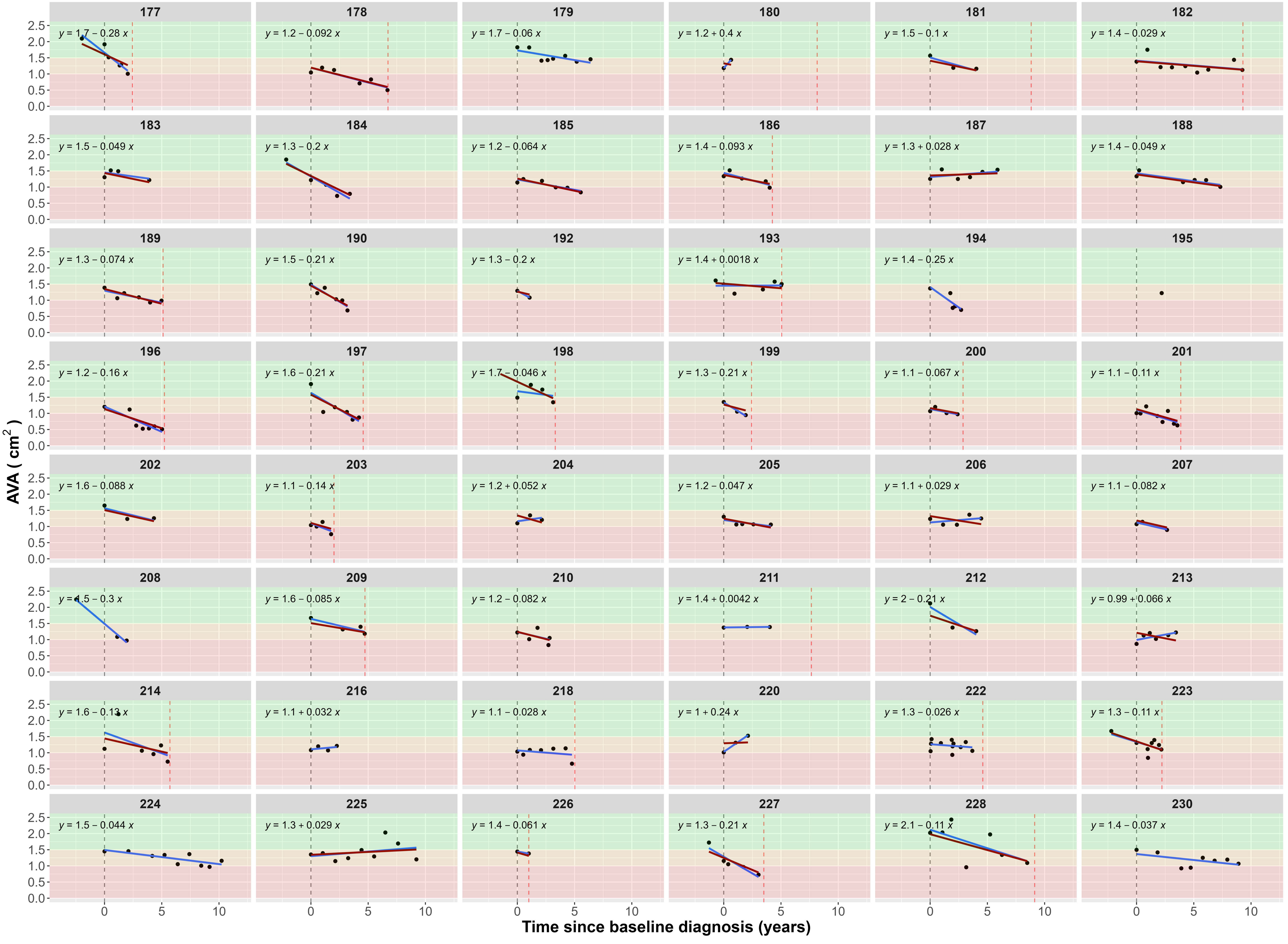

Progression of AVA (figure 5 of 12)

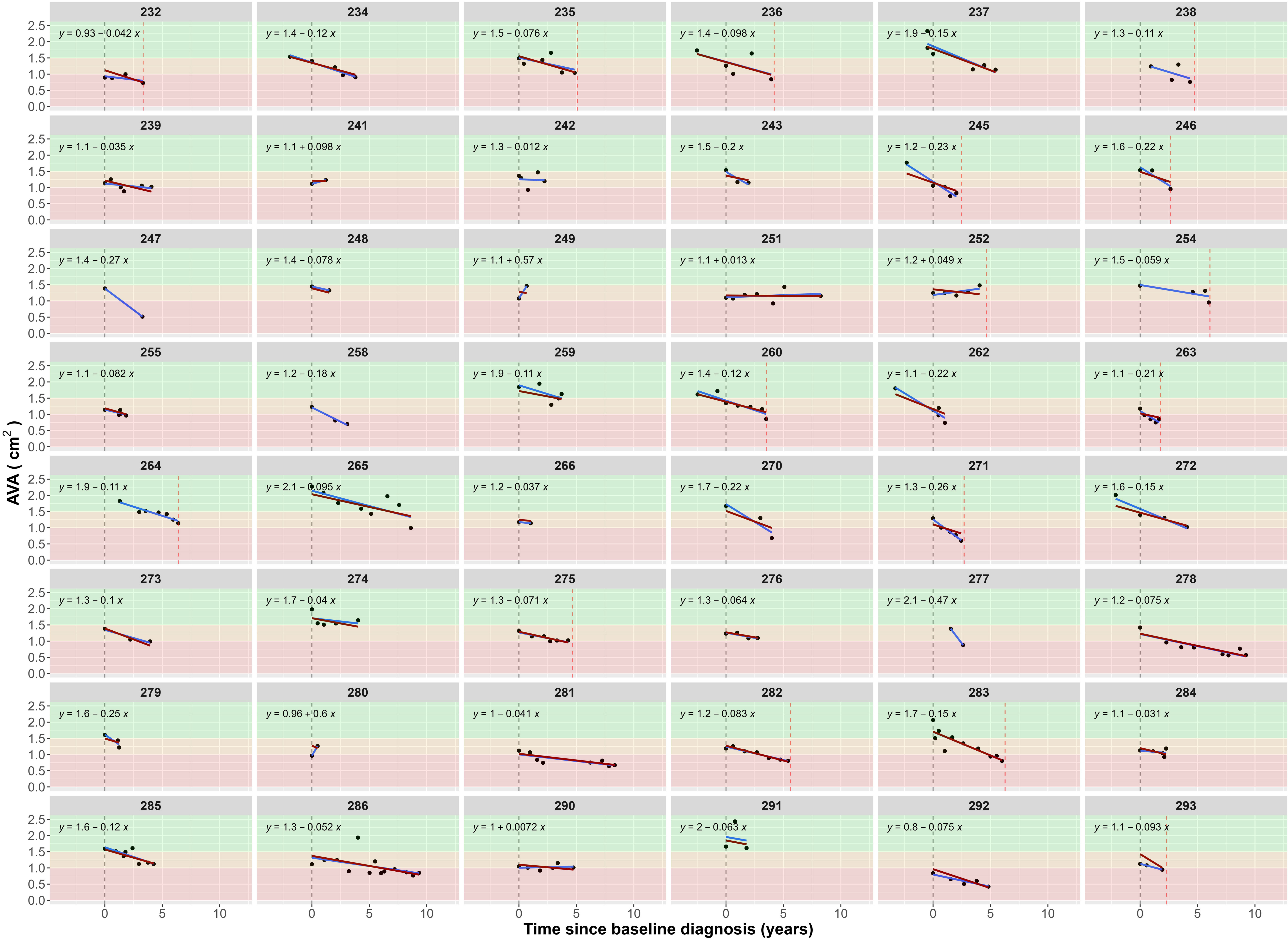

Progression of AVA (figure 6 of 12)

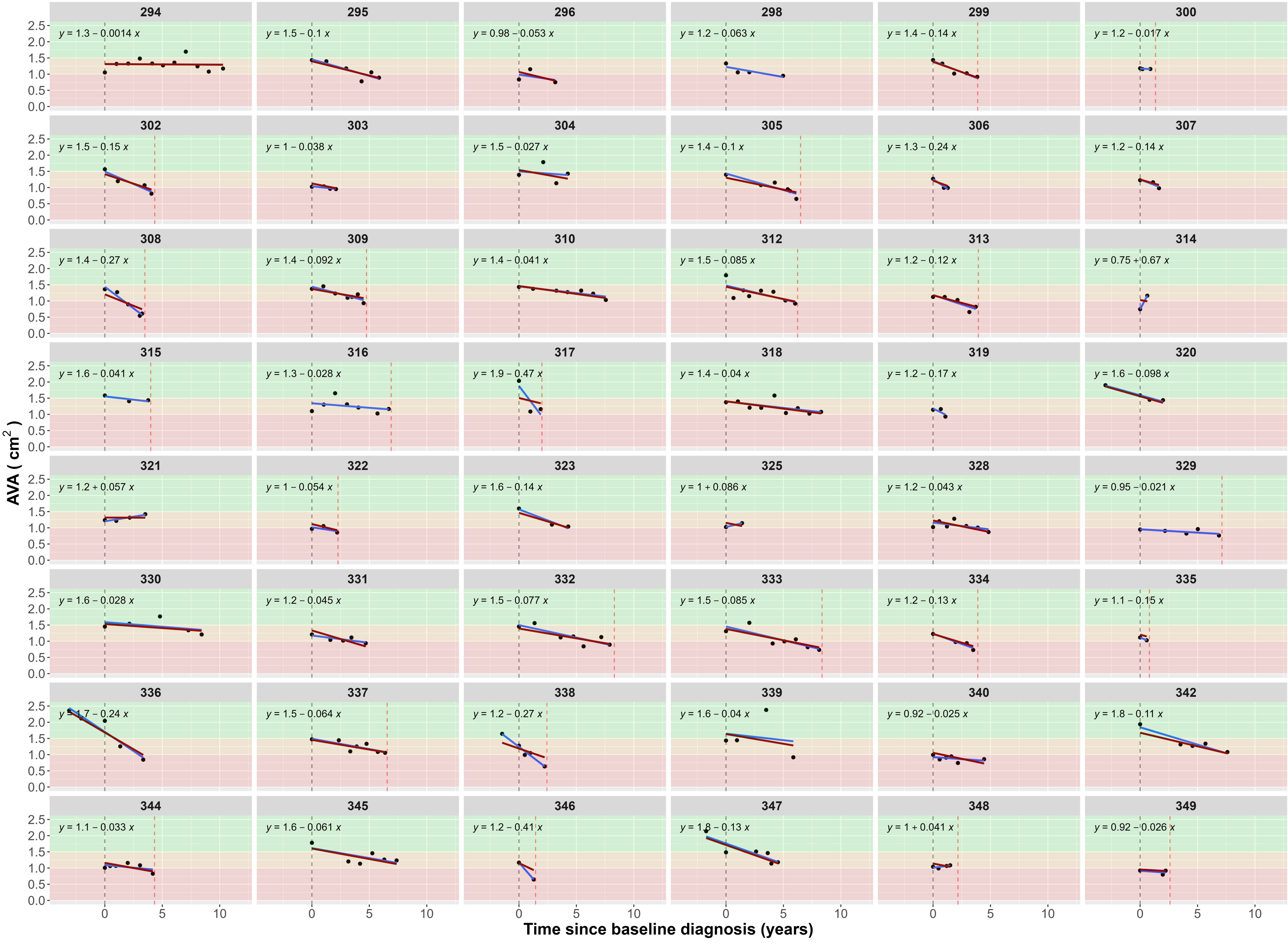

Progression of AVA (figure 7 of 12)

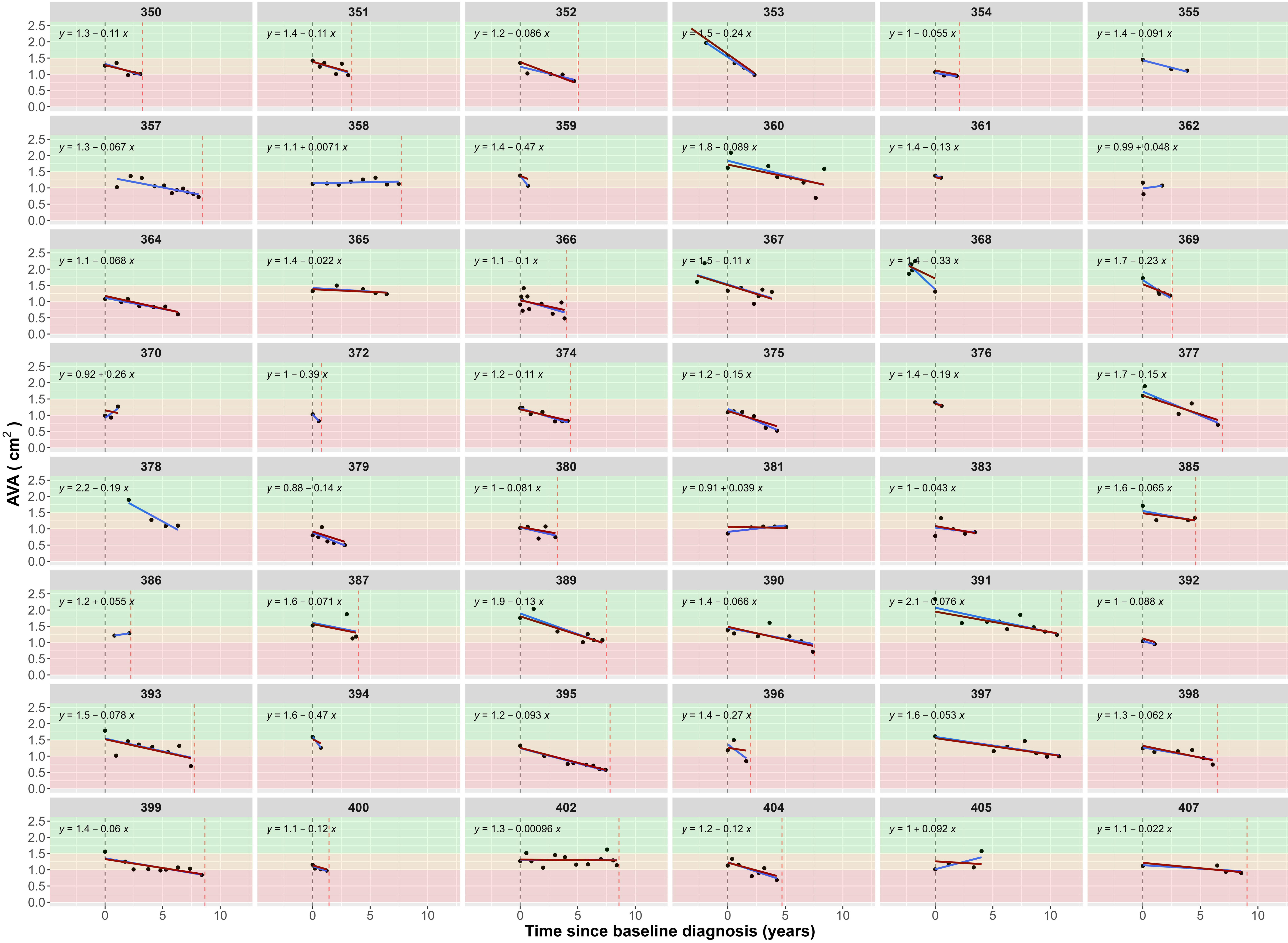

Progression of AVA (figure 8 of 12)

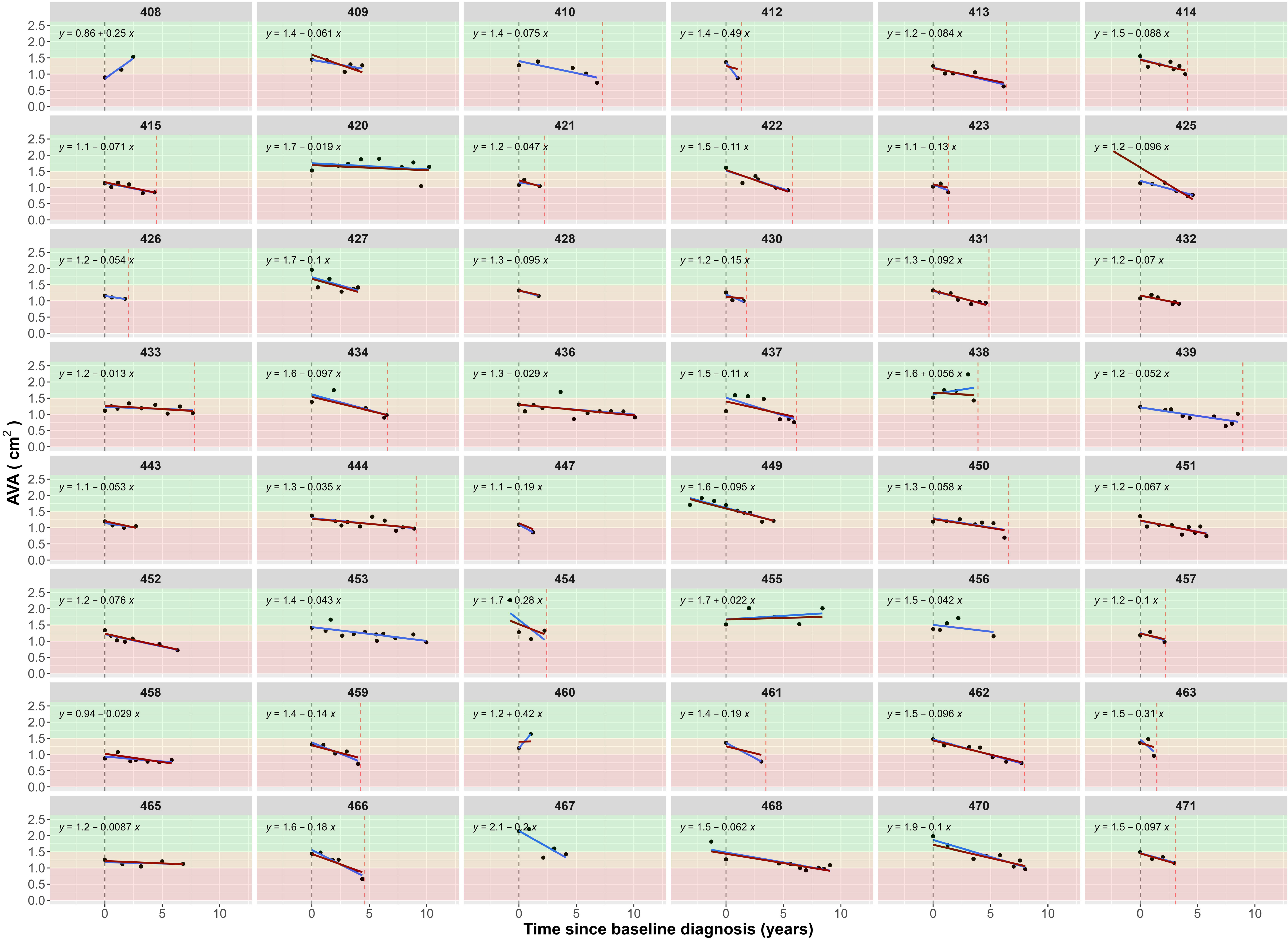

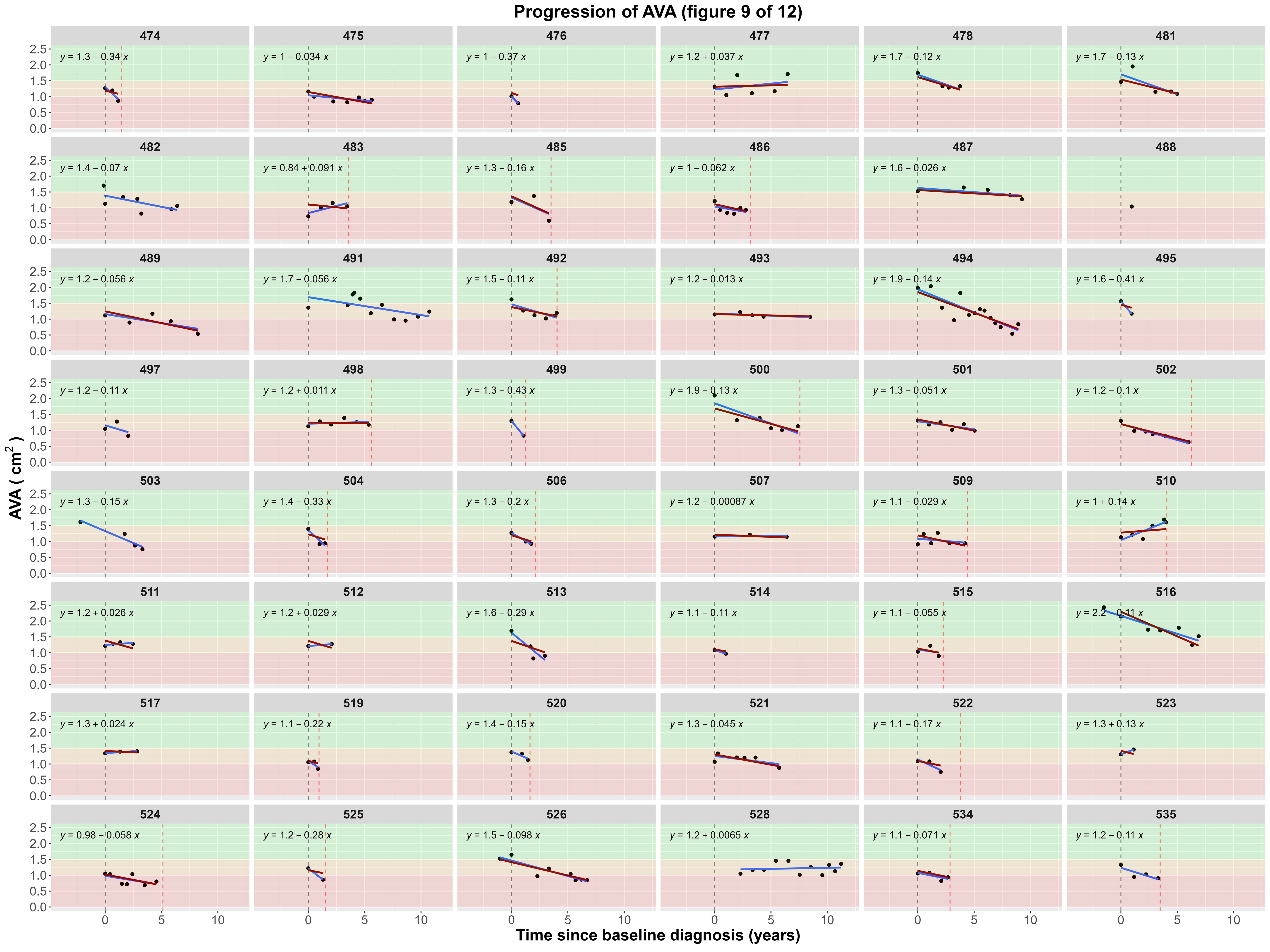

Progression of AVA (figure 9 of 12)
474
y = 1.3 − 0.34 x
475
y = 1 − 0.034 x
476
y = 1 − 0.37 x
477
y = 1.2 + 0.037 x
478
y = 1.7 − 0.12 x
481
y = 1.7 − 0.13 x
482
y = 1.4 − 0.07 x
483
y = 0.84 + 0.091 x
485
y = 1.3 − 0.16 x
486
y = 1 − 0.062 x
487
y = 1.6 − 0.026 x
488
489
y = 1.2 − 0.056 x
491
y = 1.7 − 0.056 x
492
y = 1.5 − 0.11 x
493
y = 1.2 − 0.013 x
494
y = 1.9 − 0.14 x
495
y = 1.6 − 0.41 x
497
y = 1.2 − 0.11 x
498
y = 1.2 + 0.011 x
499
y = 1.3 − 0.43 x
500
y = 1.9 − 0.13 x
501
y = 1.3 − 0.051 x
502
y = 1.2 − 0.1 x
503
y = 1.3 − 0.15 x
504
y = 1.4 − 0.33 x
506
y = 1.3 − 0.2 x
507
y = 1.2 − 0.00087 x
509
y = 1.1 − 0.029 x
510
y = 1 + 0.14 x
511
y = 1.2 + 0.026 x
512
y = 1.2 + 0.029 x
513
y = 1.6 − 0.29 x
514
y = 1.1 − 0.11 x
515
y = 1.1 − 0.055 x
516
y = 2.2 − 0.11 x
517
y = 1.3 + 0.024 x
519
y = 1.1 − 0.22 x
520
y = 1.4 − 0.15 x
521
y = 1.3 − 0.045 x
522
y = 1.1 − 0.17 x
523
y = 1.3 + 0.13 x
524
y = 0.98 − 0.058 x
525
y = 1.2 − 0.28 x
526
y = 1.5 − 0.098 x
528
y = 1.2 + 0.0065 x
534
y = 1.1 − 0.071 x
535
y = 1.2 − 0.11 x
AVA (cm2)
0.0
0.5
1.0
1.5
2.0
2.5
0
5
10
Time since baseline diagnosis (years)

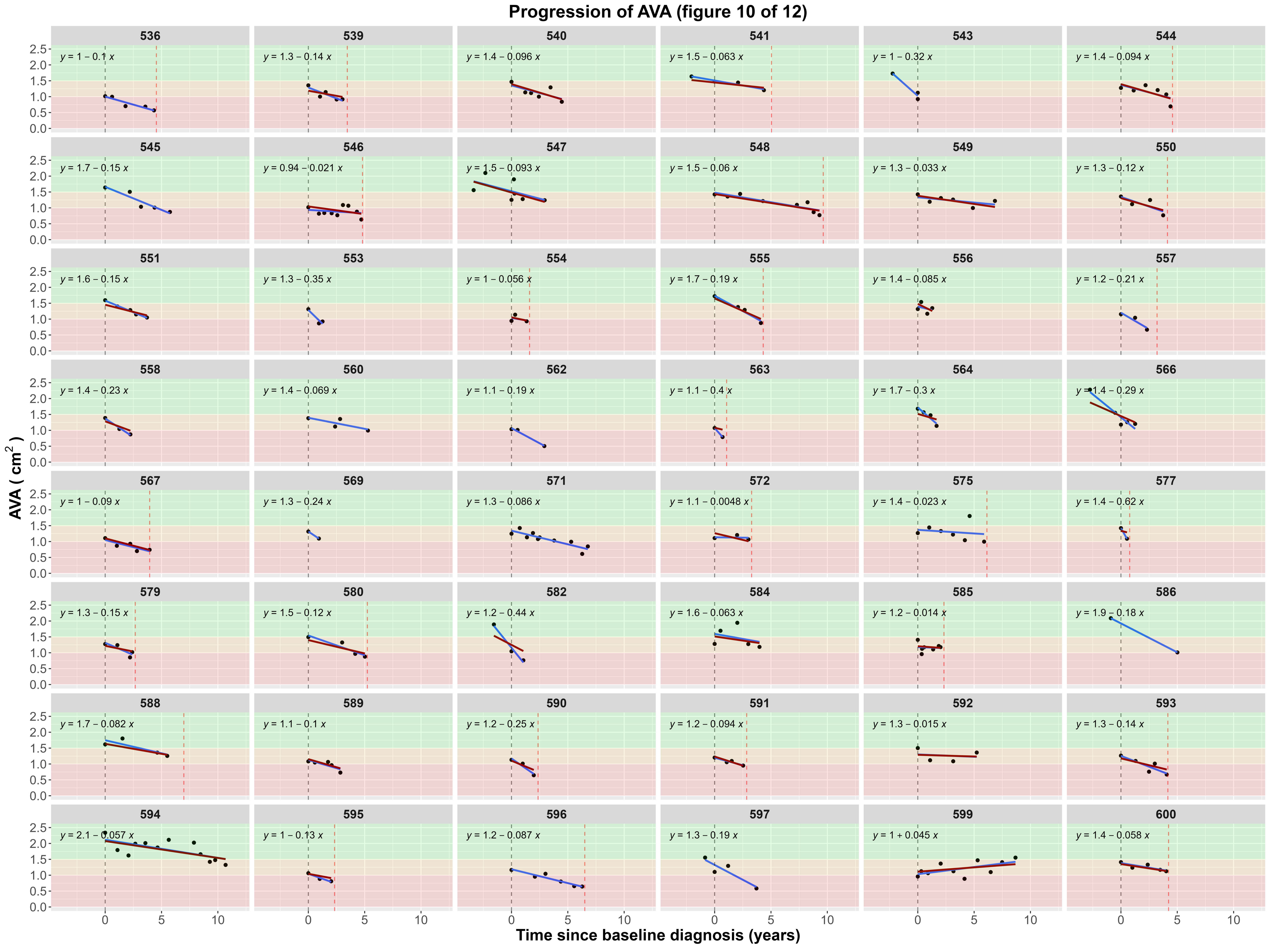
Progression of AVA (figure 10 of 12)
536
y = 1 − 0.1 x
539
y = 1.3 − 0.14 x
540
y = 1.4 − 0.096 x
541
y = 1.5 − 0.063 x
543
y = 1 − 0.32 x
544
y = 1.4 − 0.094 x
545
y = 1.7 − 0.15 x
546
y = 0.94 − 0.021 x
547
y = 1.5 − 0.093 x
548
y = 1.5 − 0.06 x
549
y = 1.3 − 0.033 x
550
y = 1.3 − 0.12 x
551
y = 1.6 − 0.15 x
553
y = 1.3 − 0.35 x
554
y = 1 − 0.056 x
555
y = 1.7 − 0.19 x
556
y = 1.4 − 0.085 x
557
y = 1.2 − 0.21 x
558
y = 1.4 − 0.23 x
560
y = 1.4 − 0.069 x
562
y = 1.1 − 0.19 x
563
y = 1.1 − 0.4 x
564
y = 1.7 − 0.3 x
566
y = 1.4 − 0.29 x
567
y = 1 − 0.09 x
569
y = 1.3 − 0.24 x
571
y = 1.3 − 0.086 x
572
y = 1.1 − 0.0048 x
575
y = 1.4 − 0.023 x
577
y = 1.4 − 0.62 x
579
y = 1.3 − 0.15 x
580
y = 1.5 − 0.12 x
582
y = 1.2 − 0.44 x
584
y = 1.6 − 0.063 x
585
y = 1.2 − 0.014 x
586
y = 1.9 − 0.18 x
588
y = 1.7 − 0.082 x
589
y = 1.1 − 0.1 x
590
y = 1.2 − 0.25 x
591
y = 1.2 − 0.094 x
592
y = 1.3 − 0.015 x
593
y = 1.3 − 0.14 x
594
y = 2.1 − 0.057 x
595
y = 1 − 0.13 x
596
y = 1.2 − 0.087 x
597
y = 1.3 − 0.19 x
599
y = 1 + 0.045 x
600
y = 1.4 − 0.058 x
AVA (cm2)
0.0
0.5
1.0
1.5
2.0
2.5
0
5
10
Time since baseline diagnosis (years)

Progression of AVA (figure 11 of 12)

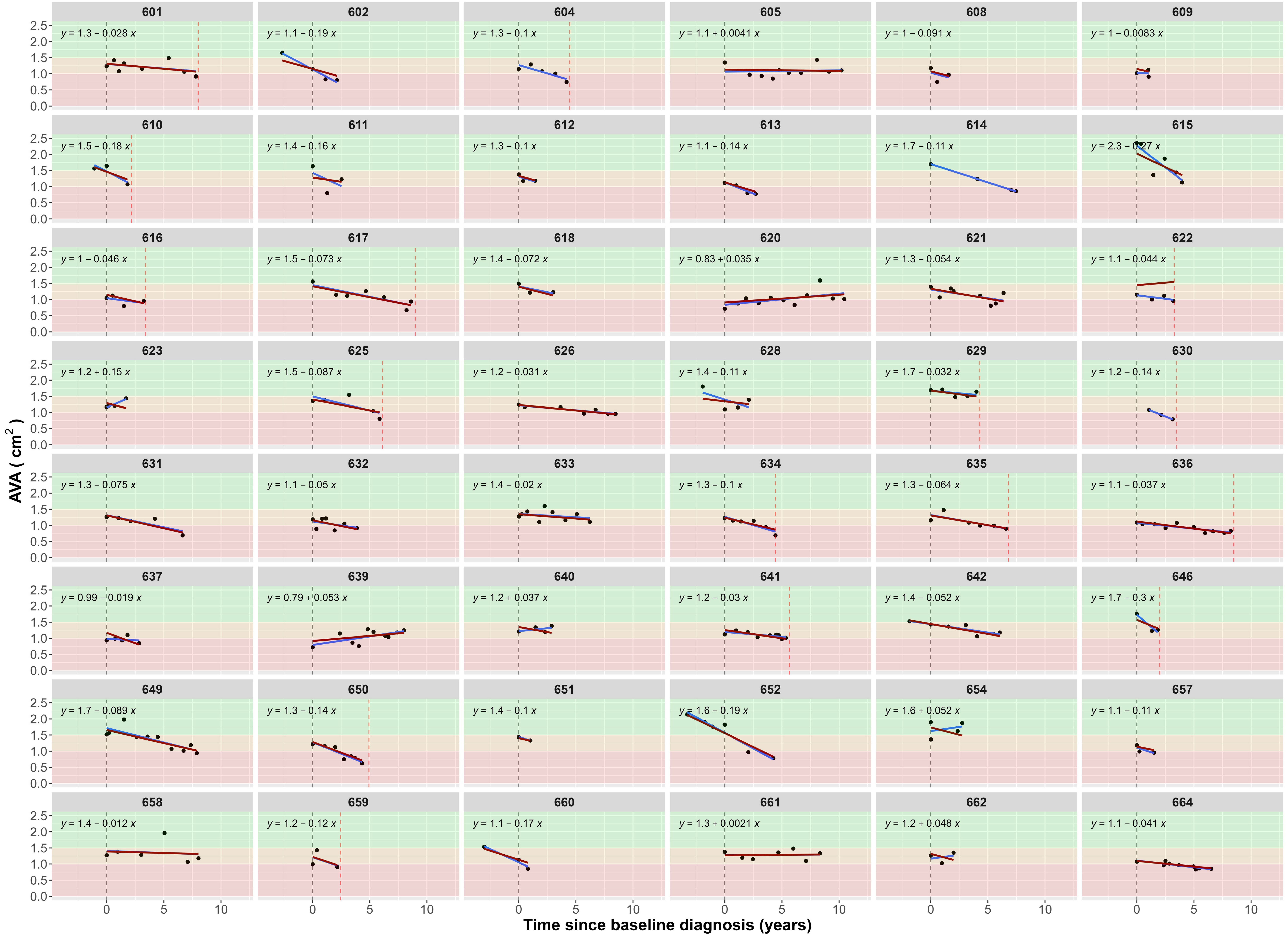

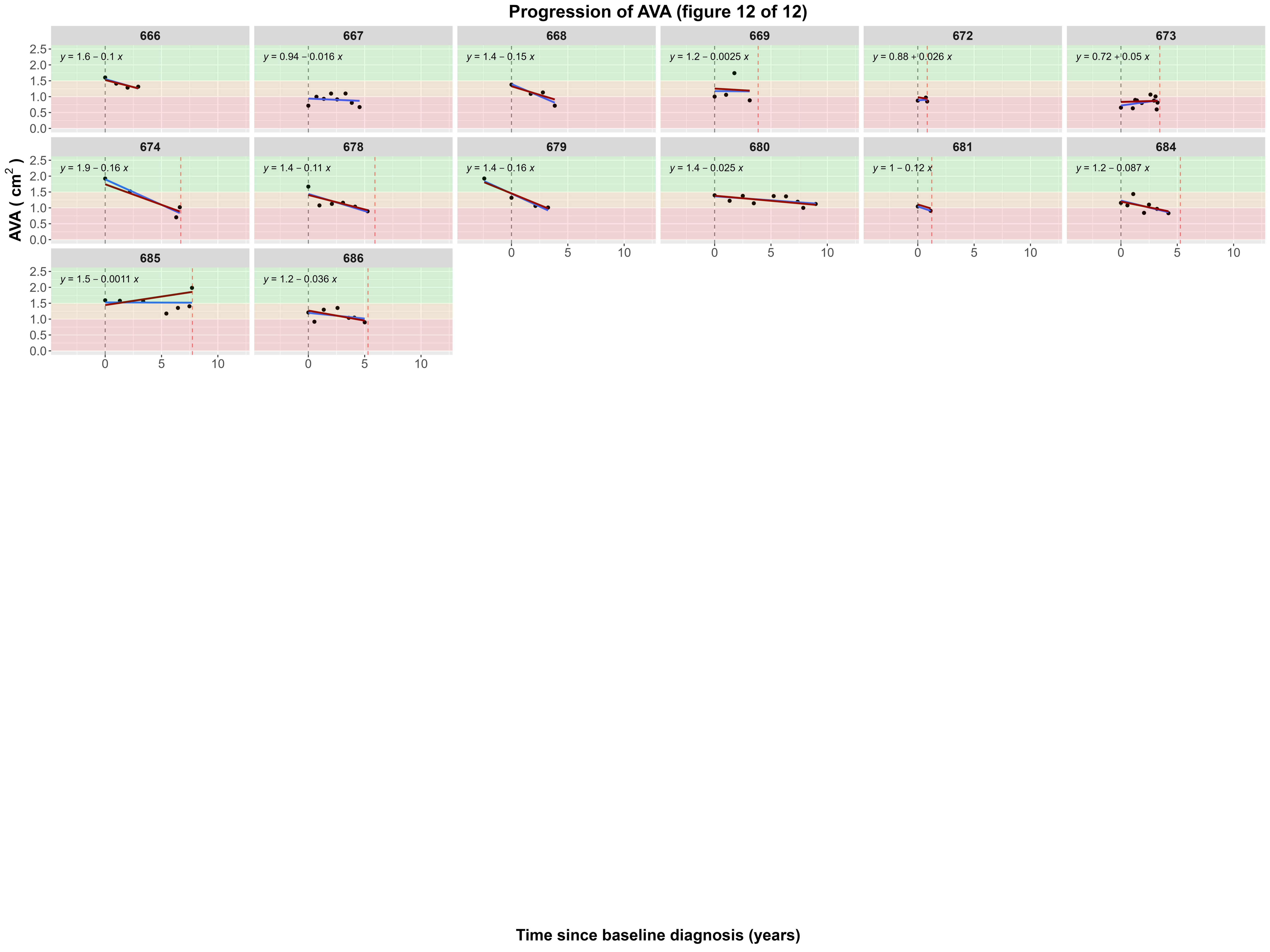

Progression of AVA (figure 12 of 12)
666
y = 1.6 − 0.1 x
667
y = 0.94 − 0.016 x
668
y = 1.4 − 0.15 x
669
y = 1.2 − 0.0025 x
672
y = 0.88 + 0.026 x
673
y = 0.72 + 0.05 x
674
y = 1.9 − 0.16 x
678
y = 1.4 − 0.11 x
679
y = 1.4 − 0.16 x
680
y = 1.4 − 0.025 x
681
y = 1 − 0.12 x
684
y = 1.2 − 0.087 x
685
y = 1.5 − 0.0011 x
686
y = 1.2 − 0.036 x
AVA (cm2)
0.0
0.5
1.0
1.5
2.0
2.5
0
5
10
Time since baseline diagnosis (years)
